# System dynamics modeling for cancer prevention and control: A systematic review

**DOI:** 10.1371/journal.pone.0294912

**Published:** 2023-12-01

**Authors:** Erin S. Kenzie, Mellodie Seater, Wayne Wakeland, Gloria D. Coronado, Melinda M. Davis

**Affiliations:** 1 OHSU-PSU School of Public Health, Oregon Health & Science University, Portland, Oregon, United States of America; 2 Systems Science Program, Portland State University, Portland, Oregon, United States of America; 3 Oregon Rural Practice-Based Research Network, Oregon Health & Science University, Portland, Oregon, United States of America; 4 Kaiser Permanente Center for Health Research, Portland, Oregon, United States of America; 5 Department of Family Medicine, Oregon Health & Science University, Portland, Oregon, United States of America; University of Nairobi, KENYA

## Abstract

Cancer prevention and control requires consideration of complex interactions between multilevel factors. System dynamics modeling, which consists of diagramming and simulation approaches for understanding and managing such complexity, is being increasingly applied to cancer prevention and control, but the breadth, characteristics, and quality of these studies is not known. We searched PubMed, Scopus, APA PsycInfo, and eight peer-reviewed journals to identify cancer-related studies that used system dynamics modeling. A dual review process was used to determine eligibility. Included studies were assessed using quality criteria adapted from prior literature and mapped onto the cancer control continuum. Characteristics of studies and models were abstracted and qualitatively synthesized. 32 studies met our inclusion criteria. A mix of simulation and diagramming approaches were used to address diverse topics, including chemotherapy treatments (16%), interventions to reduce tobacco or e-cigarettes use (16%), and cancer risk from environmental contamination (13%). Models spanned all focus areas of the cancer control continuum, with treatment (44%), prevention (34%), and detection (31%) being the most common. The quality assessment of studies was low, particularly for simulation approaches. Diagramming-only studies more often used participatory approaches. Involvement of participants, description of model development processes, and proper calibration and validation of models showed the greatest room for improvement. System dynamics modeling can illustrate complex interactions and help identify potential interventions across the cancer control continuum. Prior efforts have been hampered by a lack of rigor and transparency regarding model development and testing. Supportive infrastructure for increasing awareness, accessibility, and further development of best practices of system dynamics for multidisciplinary cancer research is needed.

## Introduction

Cancer is a complex, multilevel phenomenon [1–5] in which health outcomes depend on highly interrelated elements across stages of etiology, prevention, screening, diagnosis, treatment, survivorship, and end-of-life care [6, 7]. Standard bench and clinical research approaches alone are not well suited to account for this complexity, as most cancer control interventions often focus on explicit, often linear health outcomes of a specific, well-defined population [1, 8, 9]. To overcome persistent barriers to providing high-quality, equitable care, approaches are needed to address this complexity and provide guidance for successful intervention.

In a 2019 report, the National Academies of Sciences, Engineering, and Medicine argued that systems science methods could help illustrate the multilevel nature of cancer effects and costs to society and help cancer control efforts be more agile and adaptive [7]. The Journal of the National Cancer Institute published several reports calling for the use of complex, multilevel, or systems-focused approaches to advance the landscape of cancer prevention and control [1, 2, 10]. Systems models, particularly well calibrated simulation models, provide a relatively low-cost and low-risk opportunity for comparing potential interventions or treatments *in silico* before involving busy clinics and patients in trials [1, 7, 11, 12]. The field of implementation science, which studies and promotes the uptake of evidence-based interventions and practices, has also seen a call for expanded use of systems science methods [9, 13–15].

System dynamics is a multifaceted systems science approach for analyzing the complex interrelationships between variables in a system and the resulting nonlinear system behavior [16]. The National Institutes of Health (NIH) have supported the use of system dynamics approaches in a range of areas including obesity, mental health, and workforce modeling [17–19]. System dynamics applications have also been used in areas related to upstream prevention efforts focusing on issues like nutrition, exercise, vaccinations, and tobacco control, in addition to downstream efforts in treatment and care [20–23]. Although system dynamics modeling has been utilized in various aspects of public health since the 1970s, past reviews in this field have noted a significant increase in their use since the early 2000s, especially in areas focused on chronic or non-communicable diseases [24, 25]. The extent to which system dynamics modeling has been applied specifically to cancer prevention and control has not been studied.

### System dynamics modeling

System dynamics models can take the form of computational simulation models or diagramming approaches, which include stock-and-flow diagrams or causal-loop diagrams [16]. Computational simulation models are based on ordinary differential equations and generate graphs of behavior over time for key system variables. Stock-and-flow diagrams are visual representations of the structure of simulation models that are sometimes used as a standalone diagramming approach. Causal-loop diagrams are based on the same logic, but use a notation that emphasizes the feedback loops underlying system behavior. In contrast with analytic or statistical models generated directly from data, system dynamics models are designed in a top-down or theory-driven fashion by a modeler or modeling team, ideally in collaboration with a group of stakeholders or experts [9, 16].

Systems models can help researchers and decision-makers anticipate the potential intended or unintended effects of policies, interventions, or changes in context over time [11, 26]. System dynamics modeling can incorporate the time delays and feedback loops associated with complex health issues and help identify potential leverage points to improve population health [3, 21, 24, 25]. Because these models capture how systems change over time, they are particularly applicable in cancer prevention and control [1, 7, 24].

General advantages to system dynamics modeling include its suitability to capture whole-system structures representing the factors and interdependencies influencing the performance and behavior of the system [24, 27]. System dynamics models can incorporate a broader set of boundaries, including variables that would otherwise be excluded in models relying on established empirical data sources or when data is sparse [1, 9]. These models are also well suited for modeling continuous processes and capturing information flow and feedback loops, the structure of which can be easily presented in causal-loop and/or stock-and-flow diagrams [24, 28].

Because simulation studies are operationalized with differential equations, they are capable of generating synthetic results based on specified model inputs. This functionality allows for simulation models to be validated against time series data from observation or prior validated models, which builds confidence in model structure and parameterization and provides an opportunity for correction. Simulation models that are calibrated with values, or parameters, reflecting observed measurements or informed expert opinion can be used to compare potential preventive measures, interventions, or treatment regimens with a greater deal of precision than diagram-only approaches, but generally require more extensive source data.

## Methods

This review was conducted according to PRISMA (Preferred Reporting Items for Systematic Reviews and Meta-Analysis) 2020 guidelines [29] (S1 Appendix). Scholarly articles describing the development or application of system dynamics models for cancer prevention and control were identified, abstracted, and analyzed. The data sources and search strategy were developed with the support of two health science research librarians. A formal research protocol was not created for this review. The study was not registered in the International Prospective Register of Systematic Reviews (PROSPERO) as it was a methods review and did not assess health outcomes. The review, data abstraction, quality assessment, and qualitative synthesis were conducted by three analysts with expertise in system dynamics and qualitative research (EK, MS, and WW), with any disagreements resolved through discussion.

### Search strategy

Searches for peer-reviewed publications were conducted between February and April 2022 through the PubMed, Scopus, and APA PsycInfo databases using the search terms “cancer” AND ("causal loop" OR "system dynamics*" OR "systems thinking" OR "group model*"). This search strategy was intentionally broad in order to capture all potentially relevant publications relating to cancer prevention and control, including those not specifically categorized as such within the controlled vocabularies of our search databases (e.g. HPV, smoking, nutrition, etc.). To keep a primary focus on cancer, we excluded studies related to topics such as nutrition and physical activity if they did not explicitly include cancer in their models or research aims. Additional searches were conducted in eight journals (*System Dynamics Review*, *American Journal of Public Health*, the *Journal of the Operational Research Society*, *Systems Research and Behavioral Science*, *Health Research Policy & Systems*, *BMC Health Services Research*, *Journal of the National Cancer Institute*, and *Cancer*). Selection of these journals was informed by a past review by Liu and colleagues [24], who identified a number of journals that published the most papers applying system dynamics modeling to health topics. Articles were also identified via a “snowball” method through citation and author searching. A detailed search strategy can be found in S2 Appendix.

### Review strategy

Titles and abstracts of publications identified through the searches were independently reviewed by two analysts (ES, MS) for fit to criteria. The full text of screened articles were reviewed by both analysts to ensure each article met the criteria. Included articles: 1) were published in peer-reviewed journals; 2) were written in English; 3) used system dynamics modeling (i.e., causal-loop diagramming, stock-and-flow diagramming, or computational system dynamics modeling); 4) pertained to cancer prevention or control; and 4) were published within the last 10 years. This search period was selected to reflect recent developments and current applications of system dynamics modeling related to cancer. Articles that fit the eligibility criteria were saved to Zotero, a reference management software program.

### Data abstraction and analysis

The analysts independently abstracted information pertaining to type of SD model, research aim/purpose, unit modeled, study location, information sources, model development processes, verification/validation, people involved in model development, how the model was used/applied, advantages and disadvantages of the modeling approach, and sources of funding was entered into a spreadsheet. The studies were then independently categorized by their primary focus areas in the cancer control continuum (etiology, prevention, detection, treatment, and survivorship), as presented by the National Cancer Institute [6]. Discrepancies at any point were discussed until consensus was reached.

To assess the quality of system dynamics modeling and documentation, two analysts with backgrounds in system dynamics (EK, WW) developed criteria adapted from Davahli and colleagues [30] and Rahmandad and Sterman [31] outlined in Table 1 and provided in detail in S3 Appendix. These criteria were selected to be inclusive of diagram-only and simulation approaches and relevant to models relating to cancer prevention and control. While most of the criteria pertain to both types of approaches, the expectations for diagram-only and simulation approaches within those criteria are necessarily different. For example, proper model verification and validation (Criteria 5) for simulation models involves clear documentation of model structure, equations, and parameter values and their empirical support [16]. Diagram-only studies were expected to demonstrate a process of ensuring that model structure aligns with the intended mental model or source material used to generate the model (e.g., through obtaining feedback from stakeholders) [16]. Involvement of stakeholders in model development, validation, and use (Criteria 4) was included due to the applied nature of these models to cancer prevention and control. The extent and form of stakeholder involvement varies in part according to the purpose of the model, which guides source material. A model intended to summarize findings from literature review, for example, would have more limited participatory involvement.

**Table 1:** Quality criteria for system dynamics models.

Criteria	Diagrams	Simulation
1. Present clear objectives or purpose appropriate for system dynamics	X	X
2. Identify information sources supporting model development	X	X
3. Clearly describe modeling process, including role of modeler(s) & participants	X	X
4. Involve stakeholders in model development, validation, and use, as appropriate	X	X
5. Verify and validate model	X	X
6. Describe model structure using diagram(s) adhering to standard notation	X	X
7. Calibrate the model using real-world data, as appropriate		X
8. Present clear model output and results using graphs, charts or tables		X
9. Report model equations and parameter values		X

Adapted from Davahli et al. [30] and Rahmandad and Sterman [31].

The criteria were independently applied to the included articles using the abstracted data and supplemental review of source material. Fit to each criterion was rated on a six-point scale (0–1 = missing or minimal; 2–3 = criteria partially met; 4–5 = criteria met satisfactorily). The analysts met to discuss rating discrepancies to reach consensus for different criteria ratings.

Abstracted data pertaining to model purpose, unit modeled, journal type, funding source, methods for model development and application, and author appraisal of modeling were qualitatively synthesized [11, 17, 21, 24, 32]. Due to the heterogeneity of the included studies, a narrative synthesis was conducted to describe the respective characteristics of each study.

## Results

### Characteristics of included studies

**Fig 1:**
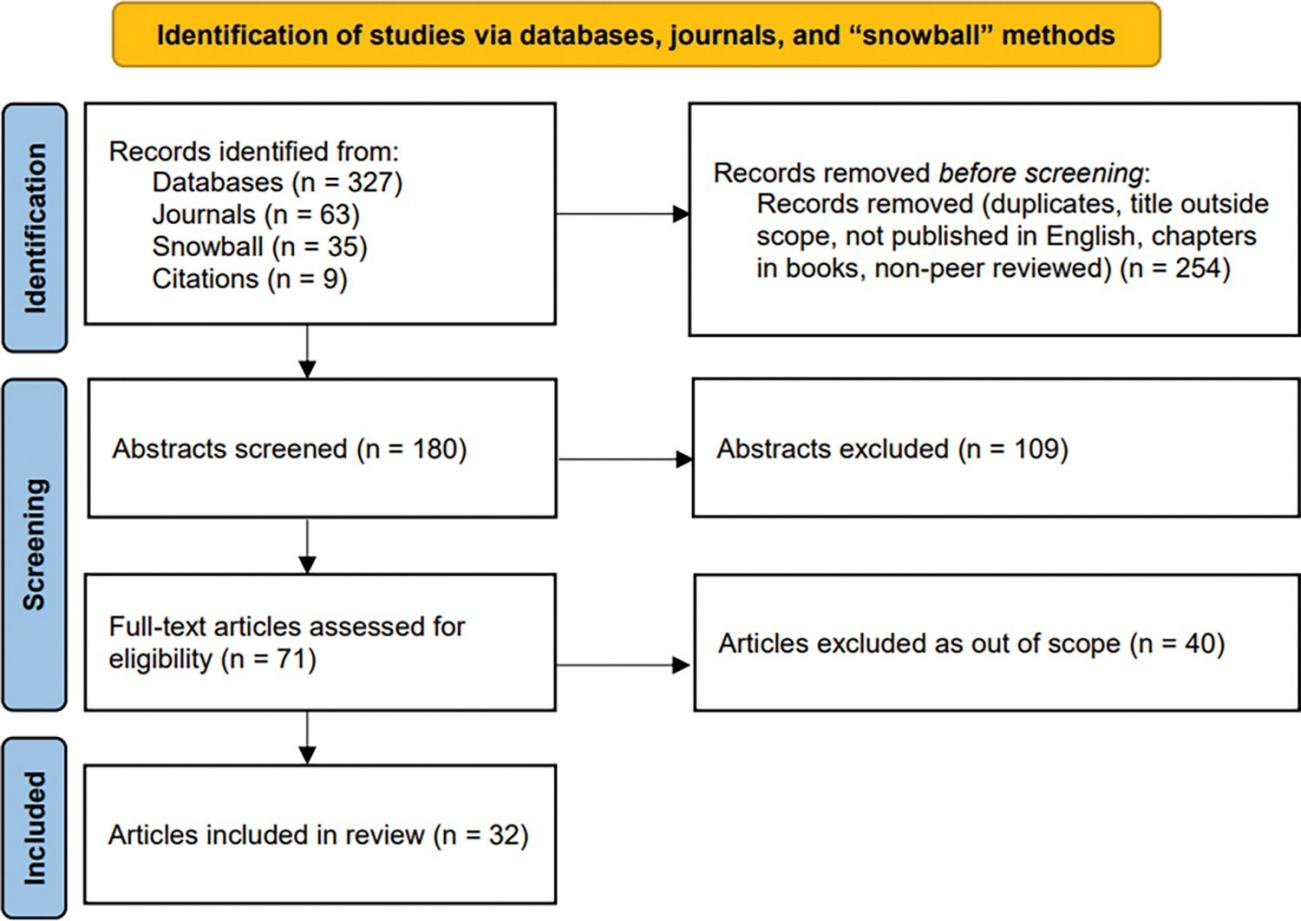
PRISMA flow diagram. PRISMA diagram of study search, review, and inclusion for review.

A total of 434 studies were retrieved and 32 studies met inclusion criteria, as seen in Fig 1. Articles that focused exclusively on cancer pathogenesis at the cellular or molecular levels were excluded, as were studies that exclusively used other types of modeling (e.g., agent-based simulation, network modeling, microsimulation, dynamical systems models). Our final set of 32 studies pertained to 14 countries and were published in 29 different journals. Three-quarters of studies (n = 25; 78%) indicated a source of funding. Only six authors were involved in more than one publication, and none were involved in more than two publications.

### Model characteristics and purpose

As shown in Fig 1, most studies included a computational system dynamics simulation model (n = 21; 66%), while the remaining studies (n = 11; 34%) used only diagramming approaches (referred to as *diagram-only* studies in this review). Of the diagram-only studies, six used causal-loop diagramming only, two used a hybrid causal-loop diagram / stock-and-flow diagram, two exclusively used stock-and-flow diagramming, and one used causal-loop diagramming in addition to a hybrid causal-loop diagram / stock-and-flow diagram. Some studies used system dynamics modeling as part of a mixed methods study in conjunction with other methods, such as mixed integer programming [33] or qualitative interviews and surveys [34]. People were the primary unit being modeled in 50% of the studies (n = 16) [34–49], while 16% modeled patient experience or behavior (n = 5) [50–54]. Other primary units modeled include cells (n = 4; 13%) [33, 55–57], environmental contaminants (n = 4; 13%) [58–61], currency (n = 2; 6%) [62, 63], and web search queries (n = 1; 3%) [64]. The abstracted characteristics of all studies included in this review are available in S1 Table.

Study topics were clustered around several themes: Eight studies (25%) focused on cancer screening, each of which focused on a different type of cancer or associated condition (e.g., Lynch syndrome, anal dysplasia). The remaining simulation studies related to global disease awareness, oncology nursing stress, cancer care pathways, and optimization of inputs for modeling patient flow. Diagram-only studies not relating to the above topics included models focused on global cancer disparities, cancer care pathways, non-communicable disease management, emergency department use or wait times, disparities in treatment initiation, and quality of life.

**Fig 2:**
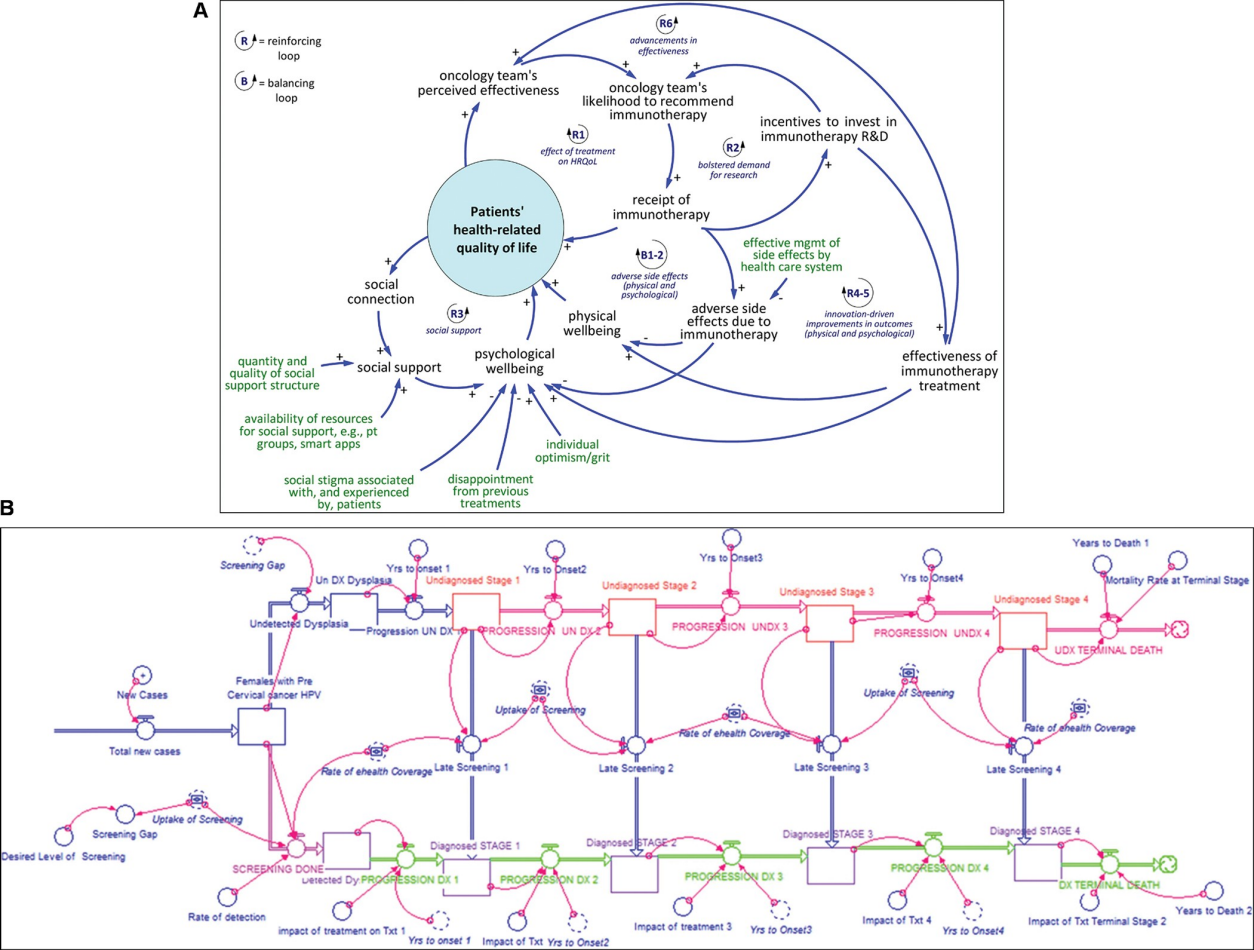
Selection of diagrams from included studies. Fig 2A shows a causal-loop diagram figure reprinted from Beaulieu and colleagues (2022) [50]. Fig 2B shows a stock-and-flow diagram figure corresponding to a simulation model reprinted from Kivuti-Bitok and colleagues (2014) [43]. All figures reproduced in accordance to Creative Commons (CC BY 4.0) license requirements.

The scope and amount of detail included in the models varied considerably. Two studies [53, 65] included only two simple feedback loops and fewer than 10 variables in their model, while most other models were considerably more complex. Many studies included multiple diagrams to illustrate overall model structure or model subcomponents, while two studies did not include diagrams or descriptions of their model structure at all. Fig 2 illustrates examples of diagrams from two included studies, including a simplified causal-loop diagram related to cancer immunotherapy and patient quality of life [50], as well as a stock-and-flow diagram corresponding to a simulation model of a Human Papillomavirus (HPV) vaccination program [43].

**Fig 3:**
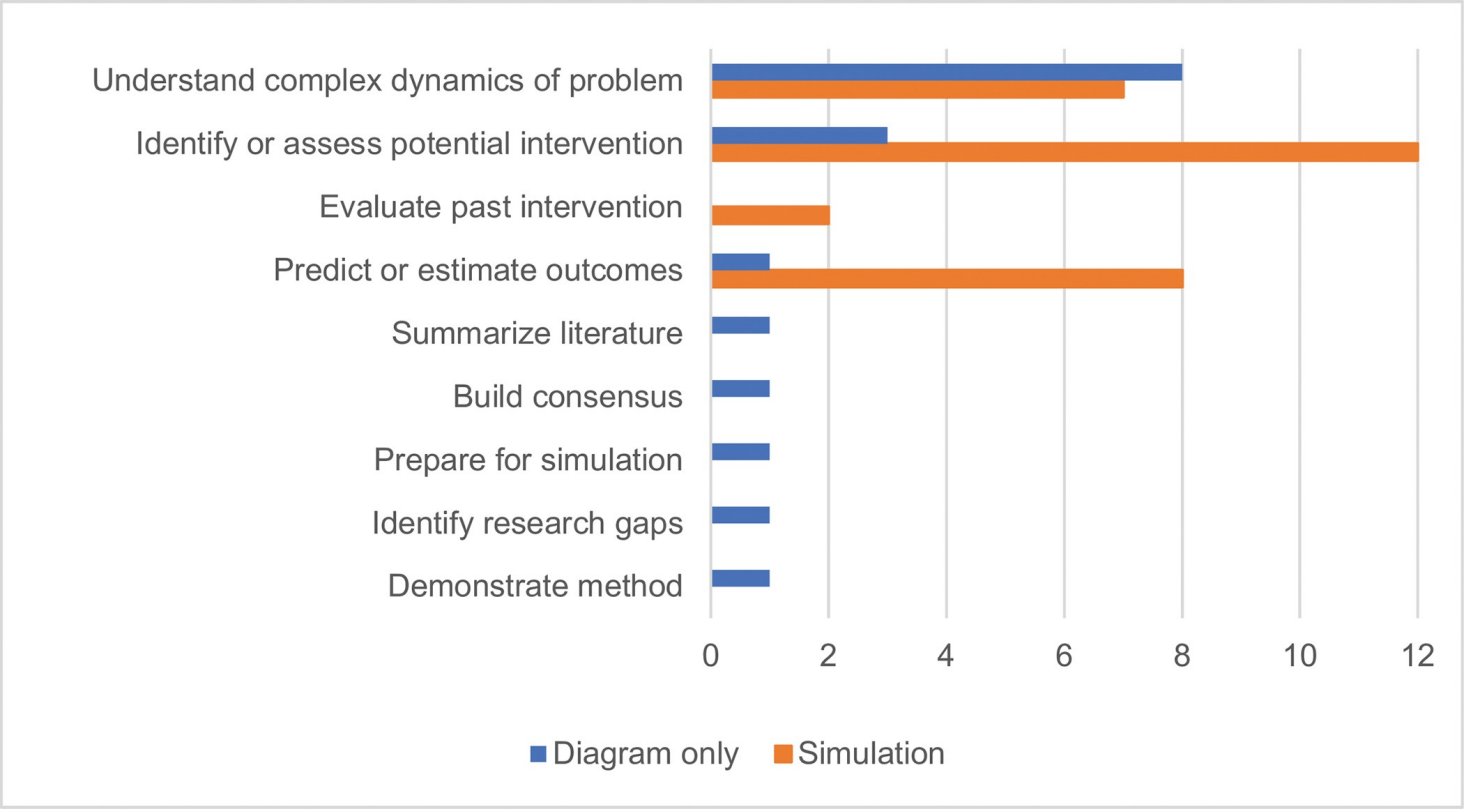
Purpose of modeling in the included studies. Number of diagram-only and simulation studies are shown by purpose (total n = 32).

As shown in Fig 3, the purpose of models developed in the included studies spanned understanding complex dynamics of the problem, identifying or assessing potential interventions, evaluating a past intervention, predicting or estimating outcomes, summarizing the literature, building consensus, preparing for simulation, identifying research gaps, and demonstrating the potential of system dynamics as a method. Both simulation and diagram-only studies used modeling to understand the complex dynamics of a problem. Simulation studies were far more likely than diagram-only studies to use modeling to identify or assess potential interventions or to predict or estimate outcomes. Studies that only used diagrams showed more diversity in purposes. Most studies of both types indicated more than one purpose.

### Model development

**Fig 4:**
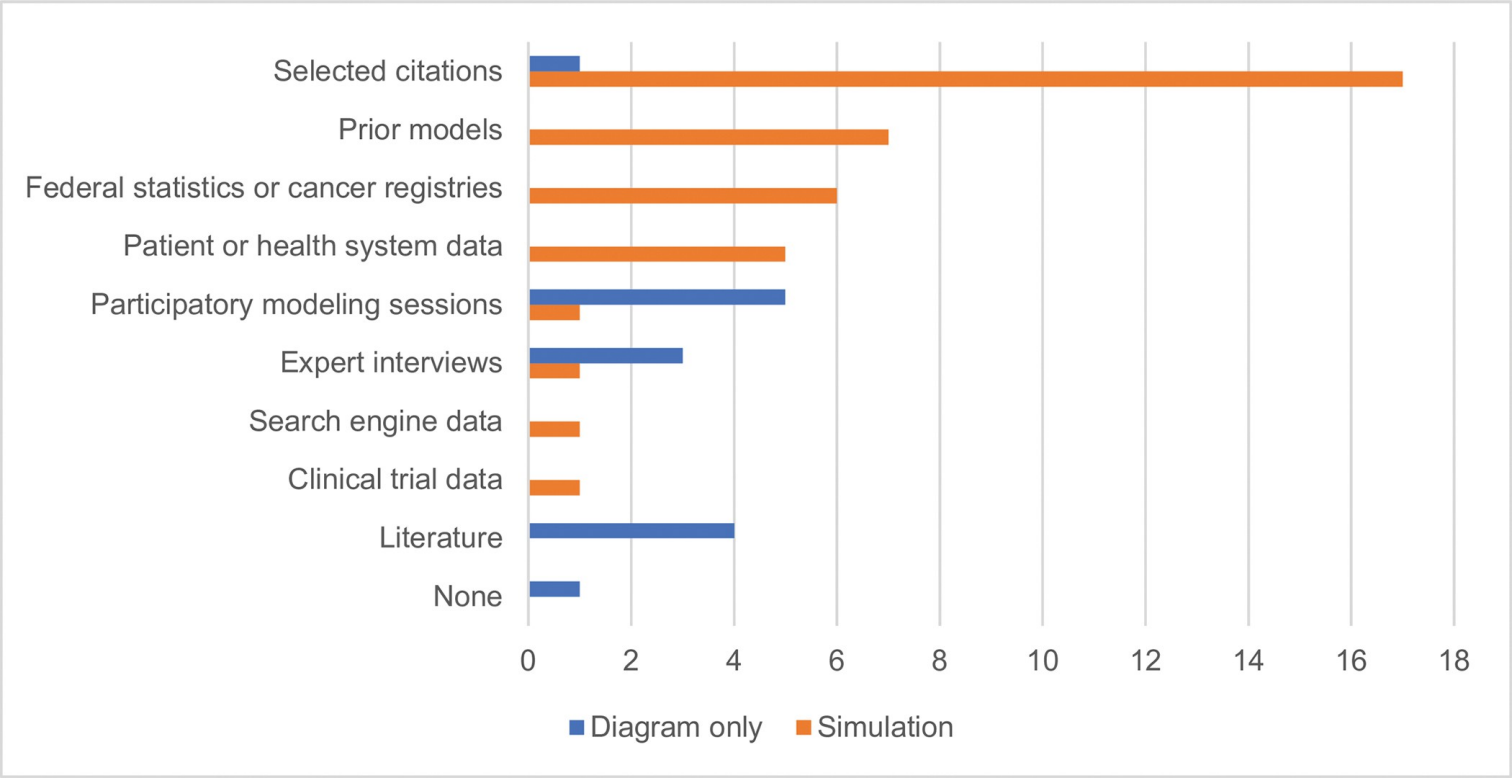
Information sources used in model development.

Information sources used for model development, illustrated in Fig 4, included citations selected by the authors (n = 18; 56%) [33, 37–44, 51, 52, 56–60, 62, 63], prior models (n = 7; 22%) [33, 34, 40, 47, 55, 58, 64], participatory group modeling sessions (n = 6; 19%) [34, 35, 49, 52–54], publicly available data from cancer registries or federal statistics (n = 7; 22%) [40, 41, 43, 44, 46, 47, 61], patient or health system data (n = 5; 16%) [34, 37, 45, 51, 53], literature review (n = 4; 13%) [36, 48, 50, 62], and expert interviews (n = 4; 13%) [41, 42, 50, 51]. One study used search engine data [64] and another used unpublished data from a previously conducted clinical trial [46]. In one study that used causal-loop diagramming to illustrate the potential applications of this approach, [39]information sources were not specified. Most simulation studies (n = 16; 76%) used citations selected by the authors to support their model; for four of these studies, this was the only information source indicated. Overall, diagram-only studies were more likely than simulation studies to use participatory modeling sessions, expert interviews, or literature review. Only two simulation studies engaged participants or experts in their work through group modeling or interviews [40, 53]. No diagram-only studies utilized prior models, federal statistics or cancer registries, patient or health system data, search engine data, or clinical trial data (Fig 4).

Although all studies cited or described sources of information that informed their models, studies varied widely in regards to the level of detail provided about the processes used for model development, such as how source materials were analyzed and used to inform model structure and parameters, how modeling decisions were made and by whom, and the steps taken to revise and validate the model. The majority of simulation studies (62%) did not specify who was involved in model development, validation, or use. The majority of diagram-only studies (73%), on the other hand, specified the role of the author, research team, participants, or experts in the modeling process. For studies that did not describe model participants or processes, it is reasonable to assume that the study authors drew from their own understanding for aspects of model development not otherwise described.

### Study topics across the cancer control continuum

**Fig 5:**
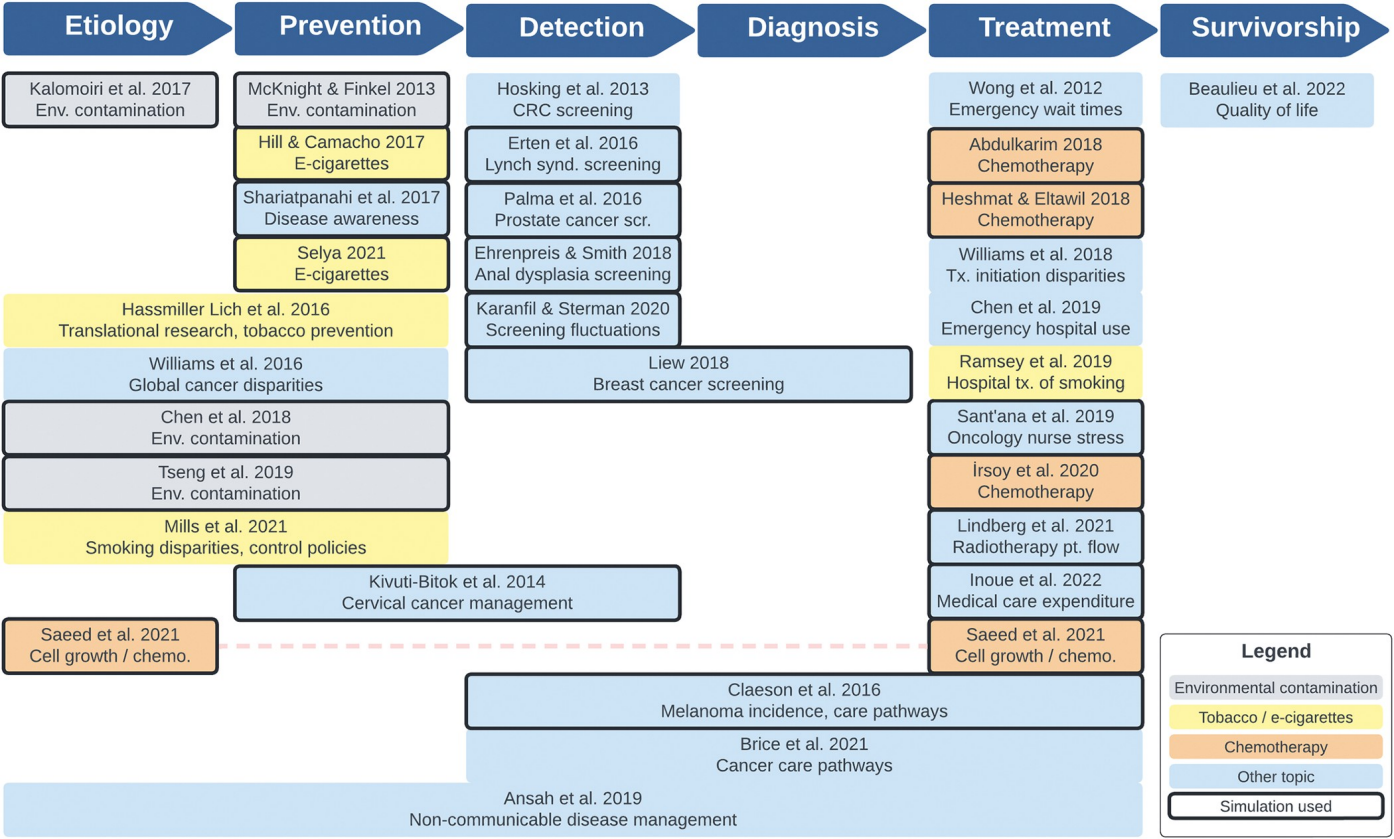
Included studies across the cancer control continuum.

As seen in Fig 5, our search yielded studies relating to all stages in the cancer control continuum: fourteen (44%) related to treatment [33–37, 45, 49, 51, 53–57, 63], eleven (34%) to prevention [35, 39, 40, 43, 47, 48, 52, 58, 60, 61, 64], ten (31%) to detection [35–38, 41–44, 46, 62], eight (25%) to etiology [35, 39, 48, 52, 57–59, 61], four (13%) to diagnosis [35–37, 51], and one (3%) to survivorship [50]. The majority of studies (n = 21; 66%) related most significantly to one focus area in the cancer control continuum, while seven (22%) related to two areas [39, 43, 44, 48, 52, 58, 61], two (6%) related to three areas [36, 37] and one (3%) related to five [35]. Studies pertaining to etiology or prevention most often related to other focus areas (typically each other), while studies related to treatment most often related to only that area. Only one study included etiology and treatment [57]. Detection was evenly split between single and multiple focus areas. No studies addressed diagnosis in isolation, while no studies addressed survivorship and another focus area. Simulation and diagram-only approaches were both used by studies in every focus area except survivorship, which only had one diagram-only study.

The majority of studies spanning etiology or prevention (62%) related to either environmental pollution or tobacco / e-cigarettes [39, 40, 47, 52, 58–61]. Five treatment studies related to chemotherapy [33, 55–57], one of which included etiology [57]. Of the ten studies discussing detection, no two related to screening for the same type of cancer. Topics of the remaining articles were heterogeneous and included cancer disparities [48, 54], treatment workflows [45, 49], healthcare service utilization or cost [51, 63], public awareness of diseases [64], and survivor quality of life [50].

### Quality assessment

Based on our application of the quality criteria outlined in Table 1, the overall quality of included studies was found to be low. Only one study [52] met all applicable criteria satisfactorily (i.e., earned a score of 4 or 5 for every criteria). Only three studies [41, 50, 54] satisfied each criteria at least partially (i.e., scored 2–5 for all criteria). The remaining 28 (88%) studies had missing or minimal components required by the quality criteria (i.e., scored 0–1 on at least one criterion).

Participant involvement and verification/validation were the criteria most likely to be missing or minimally addressed. Only four studies (13%) [34, 35, 52, 54] reported involving participants in model development, validation and use in a satisfactory way. Only one study specified that building consensus or understanding among group modeling participants was a goal of their modeling activity [35]. No simulation studies adequately described their modeling process, including the role of the modeler, while only five (16%) diagramming studies did so [35, 50–52, 54]. In total, only five studies across both modeling approaches (16%) adequately verified and validated their models [46, 50, 52, 54, 56].

**Fig 6:**
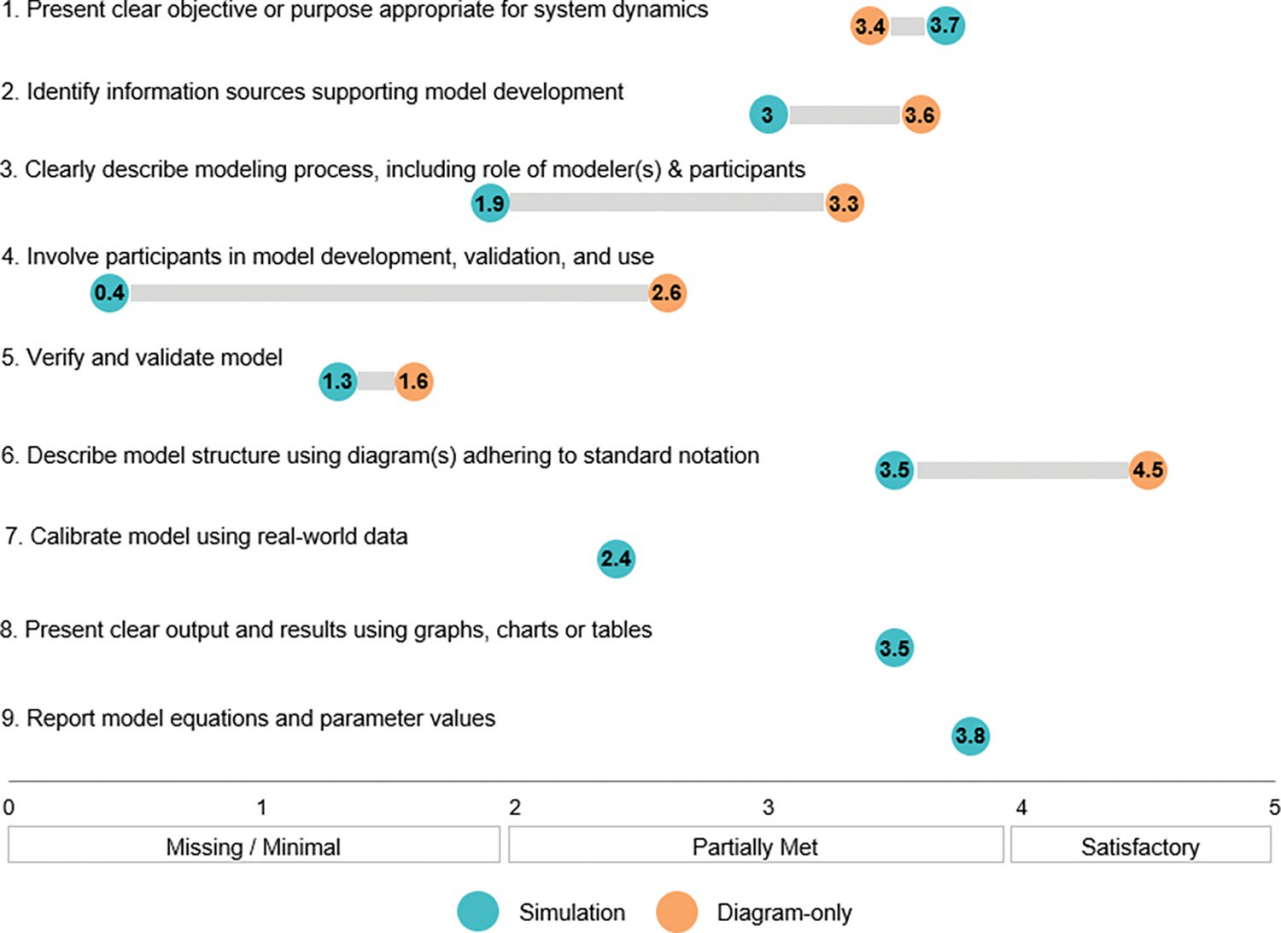
Average assessed quality score of included studies by model type.

As shown in Fig 6, the average rating for all quality assessment criteria were at an unsatisfactory level, except for one criterion (describe model structure using diagram(s) adhering to standard notation), which averaged a 4 for diagram-only studies. Quality scores of all included studies are available in S2 Table. The average quality rating of studies that used simulation was lower than studies that used only diagramming for all but one criteria that pertained to both model types (present clear objectives or purpose appropriate for system dynamics). Of the five articles that earned an overall satisfactory score, four were diagram-only articles [35, 50, 52, 54]. Simulation studies rated particularly low in participant involvement and description of model process, with none of them meeting the satisfactory threshold. Most simulation studies reported model equations and parameter values in a satisfactory way, while approximately half of them presented clear outcomes and results using graphs, charts, or tables. Many of the studies did not discuss how they verified their model structure. Only four simulation studies [40, 42, 56, 60] satisfactorily calibrated their models using real-world data or a qualitative reference behavior pattern supported by theory or expert knowledge, while model calibration was missing or minimal for seven studies. Studies that involved participants in model development, validation, and use had higher overall quality scores than those that did not. Two studies included in our review [39, 45] used system dynamics modeling primarily for the purpose of developing or demonstrating methods. In other words, their models were presented by the authors as examples and were not meant to reflect realistic dynamics. We applied the full criteria to these articles, even though methods used for such purposes might not be expected to meet the same criteria.

While diagram-only studies were rated better overall than simulation studies based on our quality criteria, overall quality was still below satisfactory. Most diagram-only studies rated below satisfactory in presentation of clear objectives or purpose, description of modeling process, participant involvement, and verification/validation. It is worth noting that validation processes for simulation studies are more extensive than for diagramming studies.

### Study appraisal of modeling

Authors of studies included in this review reported various benefits and drawbacks to using systems dynamics modeling in their studies. Several studies valued the ability of system dynamics to elucidate dynamic interactions, feedback loops, and emergent patterns of behavior across the system. Simulation studies focused on the ability to test specific scenarios like treatments or interventions, identifying and examining the effects of dynamic relationships between variables, and analyzing feedback loops. Additionally, authors of simulation models found the approach to be easily modifiable/tailorable to the research question, leaving the model open to being updated with new data as needed. Advantages to using diagramming approaches included high engagement of participants and the elicitation of valuable perspectives related to the system. Diagrams were also reportedly easy to understand and communicate, and can improve the understanding of systemic factors and drivers of feedback for those involved in the process. Nine studies (28%) suggested using diagramming methods as a decision support tool for the planning or testing of potential leverage points within the system [34, 49–55, 59]. Drawbacks were reported across both types of system dynamics modeling, and included source data limitations, the need to make assumptions/estimates when factors are outside of the scope of the system or are unknown, and difficulty representing diverse populations. Additionally, four studies (13%) did not specify any advantages or disadvantages in their publication [45, 48, 58, 64].

## Discussion

Cancer prevention and control is complex, whether looking at how to optimize chemotherapy treatment plans at the cellular level or how to increase screening at the population level. System dynamics modeling has the potential to help decision-makers and researchers find effective interventions and anticipate unintended consequences. It can also provide a platform for communities of scientists and clinicians to develop a shared understanding of challenges surrounding cancer detection, diagnosis, treatment, and survivorship. Our systematic review of studies utilizing system dynamics for cancer prevention and control yielded a wide range of applications of the method in diverse global settings. These applications demonstrate that system dynamics modeling can be used to address challenges in cancer prevention and control in ways inaccessible to standard statistical modeling. Specifically, it can be used to characterize the complexity of a problematic status quo, identify or improve interventions or strategies, and build common understanding among stakeholders. System dynamics, therefore, holds much promise for cancer prevention and control. However, our quality assessment indicates a significant opportunity to take better advantage of this method.

### Findings in context

To our knowledge, this was the first study to systematically review prior applications of system dynamics modeling across the cancer control continuum. In their review of system dynamics models pertaining to public health and medicine, Darabi and Hosseinichimeh [17] identified a handful of studies using system dynamics modeling to replicate clinical trials and simulate different interventions related to cancer, noting that this area is “generally underexplored” and would likely benefit from further development of models for cancer screening and treatment. Similar to the findings of our paper, this review found that only a fraction of disease-related modeling studies published their model equations and methodology for development.

In a scoping review of system dynamics studies related to addiction, Naumann and colleagues [65] found similarly that the method has been underutilized and that participant engagement should be further used in future studies. In their review of system dynamics models used to assess economic efficiency in public health, Jadeja and colleagues [11] also found underutilization of participatory approaches. In a study of system dynamics applications across business, environment, and health care domains, Zanker and colleagues [27] also found weak verification and validation of models published in articles that provided little or no methodological details outlining model development. These omissions threaten the validity of the specific models being described, as well as the perceived credibility and value of modeling as a method.

Stange and colleagues’ [1] literature review outlined global observations and opportunities for multilevel interventions across the cancer control continuum. As in our review, the authors noted a greater focus on topics around prevention and screening, as opposed to other areas along the continuum, like cancer survivorship.

### System dynamics across the cancer control continuum

Because system dynamics modeling can serve as a way to simulate or anticipate how potential interventions might change a system, it is not surprising that the most frequent use of this approach across the cancer control continuum related to prevention, detection, and treatment. Given that system dynamics modeling elucidates complex dynamics underlying observed outcomes, the number of studies addressing etiology could be seen as fewer than expected. Survivorship was clearly underrepresented in our sample, with only one study addressing this dimension of the cancer control continuum. Topics beyond tobacco / e-cigarettes, chemotherapy, and environmental contamination were also underrepresented in our sample. These areas present opportunities for future applications of system dynamics modeling across the cancer control continuum.

The studies relating to diagnosis did so as part of a broader investigation into care pathways or disease management, rather than diagnosis specifically. System dynamics could potentially be used to qualitatively characterize factors contributing to disparities in whether patients receive a timely and correct diagnosis. Robustly calibrated and validated simulation models could potentially be used as decision tools to aid in determining patient prognosis, but model structure would need to reliably correspond to the physiological, behavioral, or social dynamics underlying patient outcomes [17, 66].

Opportunities also exist for greater use of system dynamics diagrams and simulation models to support the implementation of evidence-based clinical and community interventions, particularly for prevention and detection. Because system dynamics models illustrate the pathways by which interventions act on context to produce outcomes, they can serve as theories of change to support decision-making [1, 15, 67, 68]. Powell and colleagues [13] have recommended the use of system dynamics modeling to aid the design, selection, and tailoring of implementation strategies and to help elucidate how implementation strategies engage underlying mechanisms. Luke and colleagues [9] argue that system dynamics modeling can be useful for developing and testing theories of implementation, as “virtual laboratories” for examining dissemination and implementation dynamics, as implementation for increasing engagement among participants, and as tools for communication with decision-makers. Jadeja and colleagues [11] point out that simulation could help guide allocation of limited resources for public sector interventions. While a growing number of studies mention the potential of system dynamics for implementation, little guidance exists for integrating system dynamics into existing implementation methods and frameworks.

### Alignment with system dynamics best practice

Over the past several decades, the field of system dynamics has developed norms and best practices for conducting and reporting research [30, 31, 69–71]. With several key exceptions, the studies we identified in our review largely did not align with these standards. Eighty-four percent of studies, including all of the simulation studies, failed to clearly describe the process they used to develop their models and only a few studies explicitly identified the role of the modeler(s). Because system dynamics modeling involves numerous choices to craft the structure of the model, select data sources, calibrate to real-world data, validate model output, and apply the model, transparency regarding how these steps were made and by whom is critically important for the credibility and reproducibility of the model [16, 27, 69, 72]. Studies that involved participants in model development, validation, or use were more likely to clearly describe their modeling processes, potentially because researchers who valued participant involvement were more likely to recognize the constructive nature of system dynamics models.

Calibration of simulation models to real-world data, verification/validation of model structure and behavior, and comparison of model results against real-world data not used to calibrate the model all help to build confidence that the model reflects the actual dynamics of a system [31]. Improperly designed or calibrated simulation models can mislead users about the anticipated effectiveness of potential interventions, particularly because the quantitative nature of model output can obscure the assumptions and decisions underlying it [73, 74]. Our findings showed that only 4 of 21 simulation studies (19%) reported proper calibration.

Contrary to best practice, several studies used system dynamics models for predicting specific future outcomes (as opposed to estimating the relative impacts of interventions) or to evaluate an existing intervention. Forrester, the founder of system dynamics, cautioned that the approach should be used to understand the “character and nature” of a system rather than specific timing or quantities of future system behavior [75]. System dynamics models are better used for understanding how complex interrelationships between variables result in nonlinear behavior and comparing in a more general sense how certain interventions or changes might affect this behavior [73].

### Recommendations for future research and supportive infrastructure

Increasing the caliber of modeling used for cancer prevention and control may involve making training and skill development opportunities more widely available [12], establishing avenues for researchers to connect with skilled system dynamics modelers, and standardizing reporting expectations across publishers. Domain-specific resources about the use of system dynamics modeling for cancer prevention and control researchers could provide guidance about how to match modeling approaches to research questions, design a system dynamics study, facilitate model development and testing, apply models effectively, and create useful documentation.

Because researchers using modeling within certain dimensions of the cancer continuum use modeling in similar ways and face similar challenges, opportunities exist for developing common strategies or best practice within these dimensions [1]. For example, standards could be developed regarding how best to obtain high-quality clinical time series data on cancer screening rates to be used, for example, to calibrate and validate models to better evaluate potential interventions for early detection. Strategies could also better engage patients and community members to help identify relevant contextual factors and potentially effective prevention interventions. Our review points to several opportunities for improving the quality of future applications of system dynamics modeling to cancer prevention and control, as well as the accessibility of system dynamics to new researchers and audiences, described in Table 2.

**Table 2:** Opportunities to advance use of system dynamics modeling for cancer prevention and control.

*Provide opportunities for system dynamics training and skill development*.	Ways to enable researchers to add system dynamics to their toolbox include increasing awareness of the types of questions system dynamics can address, the data required for calibration and validation, best practices for model development and use, prior models, and how modeling can best be integrated into existing research programs.
*Facilitate connections between cancer researchers and skilled modelers*.	System dynamics modeling is an established but relatively niche approach that requires expertise to execute effectively. Platforms for connecting researchers with expert modelers would enable productive partnerships and improve the quality of future modeling work.
*Develop best practice for modeling across the cancer control continuum*.	Tailored guidance for design of modeling studies, model development, and use for each dimension of the cancer control continuum would enable communities of researchers to better understand how to utilize system dynamics modeling.
*Encourage participatory approaches to model development*, *validation and use*.	Patients, clinic staff and providers, and community members can be valuable sources of knowledge for model structure and parameterization [76]. Participatory approaches also provide opportunities for development of shared understanding between individuals involved, which can set the stage for implementation of interventions in clinical or community settings.
*Provide a supportive funding environment*.	System dynamics modeling is often exploratory in nature, but requires expertise and resources to be done well. Development of funding mechanisms that support development of high-quality models through literature synthesis, participant engagement, and use of local data would provide opportunities for robust modeling research. Long-term funding would enable development of ‘living models’ that synthesize and communicate findings from scientific literature [77].
*Refine and make accessible model quality criteria*.	Further refinement and testing of quality assessment criteria would benefit future systematic reviews of studies using system dynamics modeling and provide guidance for future modeling studies. For example, criteria specifying appropriateness of the methods used, measurement of impact, model parsimony, and how well the aims were achieved could be included. A rubric offering more specific guidance for each criteria could also be developed.

### Limitations

This study has several limitations. In bounding our review to studies published in the last 10 years, we excluded older studies that could have contributed to this synthesis. We also excluded studies related to topics such as nutrition and physical activity if they did not include cancer in their models or research aims. While we feel this narrowed criteria was helpful in maintaining focus, inclusion of models more tangentially related to cancer might have provided a more comprehensive understanding of the current range of research. Due to our focus on cancer prevention and control, we also excluded studies that were solely related to cancer pathogenesis; if these were included, far more studies would be categorized as relating to etiology in the cancer control continuum. Our understanding of the applications of modeling covered in the included studies was also limited by the descriptions included in the articles themselves, which were shaped by journal audience. Our quality ratings, therefore, reflected both modeling and reporting. Finally, the diversity of model purposes, types, and applications presented a challenge to adapting the quality assessment criteria. While we chose to apply a universal set of criteria to included studies to enable comparison, future research could further specify tailored criteria (e.g., based on model purpose).

## Conclusion

System dynamics modeling has the potential to advance research on cancer prevention and control by synthesizing multiple kinds of knowledge and data sources to facilitate a holistic understanding of the associated complex systems and identifying potential leverage points for improving patient care. However, our systematic review of recent work in this area shows that this promise has not yet realized its full potential. Using quality assessment criteria adapted from prior literature, we found overall quality of included studies to be lacking. While we found examples of system dynamics modeling across the entire cancer control continuum, several areas–particularly survivorship–were underrepresented. To ensure accuracy and credibility, future work in this area should uphold high standards of quality for model development and reporting, including involvement of participants in model design, validation, and use. Increasing training opportunities, communication between researchers and modelers, development of best practice, enhanced funding opportunities, and establishing modeling criteria would also support successful application of system dynamics modeling for cancer prevention and control.

## Supporting information

S1 Appendix Completed PRISMA 2020 checklist. (DOCX)

S2 Appendix Search strategy. (DOCX)

S3 Appendix Quality criteria. (DOCX)

S1 Table Characteristics of studies included in review. (DOCX)

S2 Table Quality assessment scores for included studies. (DOCX)
