# Supplementary material for: System dynamics modeling for cancer prevention and control: A systematic review
Source: PLoS One. 2023 Dec 1;18(12):e0294912. doi: 10.1371/journal.pone.0294912 (PMC10691687; doi:10.1371/journal.pone.0294912)
Supplement: S2 Appendix — (DOCX) [file pone.0294912.s002.docx]

**S2 Appendix – Search strategy**

All searches were conducted between February 8 and April 1, 2022.

**Database searches:**

**PubMed**

"cancer" AND ("causal loop" OR "system dynamics*" OR "systems thinking" OR "group model building") AND (y_10[Filter])

Results: 193

**Scopus**

( TITLE-ABS-KEY ( "cancer" )  AND  TITLE-ABS-KEY (( "causal loop"  OR  "system dynamics*"  OR  "systems thinking"  OR  "group model building")))  AND  PUBYEAR  >  2012

Results: 124

**APA Psychinfo**

("cancer") AND ("causal loop" OR "system dynamics*" OR "systems thinking" OR "group model building")

Filters: 2012-2022, English, scholarly journal articles

Results: 10

**Journal searches:**

**Systems Research and Behavioral Science**

“cancer” AND ("causal loop" OR "system dynamics*" OR "systems thinking" OR "group model building")

Filters: 2012-2022

Results: 18

**Health Research Policy & Systems**

“cancer” AND ("causal loop" OR "system dynamics*" OR "systems thinking" OR "group model building")

Filters: 2012-2022

Results: 18

**BMC Health Services Research**

“cancer” AND ("causal loop" OR "system dynamics*" OR "systems thinking" OR "group model building")

Filters: 2012-2022

Results: 23

**Journal of the National Cancer Institute**

“cancer” AND ("causal loop" OR "system dynamics*" OR "systems thinking" OR "group model building")

Filters: 2012-2022

Results: 4

**Cancer**

("causal loop" OR "system dynamics*" OR "systems thinking" OR "group model building")

Filters: 2012-2022

Results: 0

**Snowball searches:**

**System Dynamics Review**:

“Cancer”

Filters: 10 years

Results: 7

**American Journal of Public Health**

("cancer") ( "causal loop"  OR  "system dynamics*"  OR  "systems thinking"  OR  "group model building")

Filters: 2012-2022

Results: 17

**The Journal of the Operational Research Society**

"cancer" AND "system dynamics"

Filters: 2012-2022

Results: 11
