# Supplementary material for: System dynamics modeling for cancer prevention and control: A systematic review
Source: PLoS One. 2023 Dec 1;18(12):e0294912. doi: 10.1371/journal.pone.0294912 (PMC10691687; doi:10.1371/journal.pone.0294912)
Supplement: S3 Appendix — (DOCX) [file pone.0294912.s003.docx]

**S3 Appendix. Quality criteria for system dynamics models**

| Criteria | 0 | 1 | 2 | 3 | 4 | 5 |
| --- | --- | --- | --- | --- | --- | --- |
| 1. Present clear objectives or purpose appropriate for system dynamics | Objectives or purpose not described and/or not appropriate for system dynamics | Very little information about objectives or purpose provided and/or not appropriate | Description of objectives or purpose inadequate and/or barely appropriate | Description of objectives or purpose only somewhat adequate or appropriate | Objectives or purpose described but not wholly appropriate (e.g., too broad) | Objectives or purpose clearly described and appropriate for system dynamics |
| 1. Identify information sources supporting model development | No mention of information sources | Very little information about modeling sources provided | Information about modeling sources partial but inadequate | Some literature identified but not associated with model components | Information sources described but not as clearly or completely as possible | Information sources clearly described |
| 1. Clearly describe modeling process, including role of modeler(s) & participants | No mention of modeling process | Very little information about modeling process provided | Description of modeling process insufficient or not adequate | Description of modeling process partial but not adequate | Modeling process described but not as clearly or completely as possible | Modeling process, including role of modelers and participants, clearly described |
| 1. Involve stakeholders in model development, validation, and use, as appropriate | No mention of stakeholder involvement | Very little information about stakeholder involvement or involvement not appropriate | Involvement of stakeholders in model development, validation, and use not well described or sufficient | Involvement of stakeholders in model development, validation, and use partly described or sufficient | Involvement of stakeholders in model development, validation, and use mostly described and sufficient | Involvement of stakeholders in model development, validation, and use clearly described and sufficient |
| 1. Verify and validate model | No mention of model verification or validation | Very little information about verification and/or validation provided | Verification and/or validation mentioned but not appropriate | Verification and/or validation procedures described but not sufficient | Verification and validation procedures adequately described and generally appropriate | Verification and validation procedures appropriate and clearly described |
| 1. Describe model structure using diagram(s) adhering to standard notation | Model structure diagrams not included | Diagrams included but not appropriate | Diagrams included but incomplete | Diagrams of model structure included but not sufficient | Diagrams describing model structure nearly adequate | Diagrams adhere to standard notation and clearly describe model structure |
| 1. Calibrate the model using real-world data / reference behavior over time | No mention of model calibration | Very little information about model calibration | Model calibration not well described or appropriate | Model calibration incomplete or limited | Model calibration adequately described and performed | Model calibration appropriate and clearly described |
| 1. Present clear model output and results using graphs, charts or tables | No graphs, charts, or tables of model output included | Graph, chart, or table included but not appropriate | Graphs, charts, or tables not adequate to describe model output and results | Graphs, charts, or tables partly adequate to describe model output and results | Graphs, charts, or tables nearly adequate to describe model output and results | Model output and results clearly described using graphs, charts, or tables |
| 1. Report model equations and parameter values | No model equations or parameters included | Up to a few model equations or parameters included | Description of model equations and parameters not sufficient | Partial description of model equations and parameters | Description of model equations and parameters nearly adequate | Model equations and parameter values clearly described |
