# Supplementary material for: System dynamics modeling for cancer prevention and control: A systematic review
Source: PLoS One. 2023 Dec 1;18(12):e0294912. doi: 10.1371/journal.pone.0294912 (PMC10691687; doi:10.1371/journal.pone.0294912)
Supplement: S1 Table — (DOCX) [file pone.0294912.s004.docx]

**S3 Table – Characteristics of studies included in review**

| **Author/date** | **Country** | **Model purpose** | **Model types** | **Information sources** | **Unit modeled** | **Participants involved** |
| --- | --- | --- | --- | --- | --- | --- |
| Abdulkarim, 2018 | Saudi Arabia | Optimize chemotherapy initiation | Simulation | Prior model | Cells | Author |
| Ansah et al., 2019 | Cambodia | Consensus among stakeholders about non-communicable disease management | Stock-and-flow diagram | Participatory modeling sessions | People | Research team, participants |
| Beaulieu et al., 2022 | International collaboration | Understand system surrounding cancer immunotherapy patients’ health-related quality of life | Causal-loop diagram | Literature review, expert interviews | Patient experience or behavior | Research team, experts |
| Brice et al., 2021 | United Kingdom | Identify factors, literature gaps, and strategies related to cancer care pathways | Causal-loop diagram | Literature review | People | Not specified |
| Chen et al., 2018 | Taiwan | Assess level of human exposure of polycyclic aromatic hydrocarbons; evaluate reduction strategies | Simulation | Prior model, selected citations | Environmental contaminants | Not specified |
| Chen et al., 2019 | United Kingdom | Understand complexity of emergency hospital use in cancer patients | Causal-loop diagram | Expert interviews | Patient experience or behavior | Research team |
| Claeson et al., 2016 | Sweden | Predict melanoma incidence, model how delays affect care pathways | Simulation | Patient data, selected citations | People | Research team |
| Ehrenpreis and Smith, 2018 | USA | Estimate anal histologic outcomes in cervical cancer patients, examine screening effects and costs | Simulation | Selected citations, health system data | People | Not specified |
| Erten et al., 2016 | USA | Compare screening strategies for Lynch syndrome | Simulation | Selected citations, health system data | Currency | Research team |
| Hassmiller Lich et al., 2016 | USA | Illustrate how system dynamics can be used across translational research stages, using tobacco control as an example | Hybrid CLD/SFD | Not specified; example models | People | Research team |
| Heshmat and Eltawil, 2018 | Egypt | Provide a decision support tool for oncologists to plan chemotherapy treatment | Causal-loop diagram, simulation | Selected citations, prior models | Cells | Not specified |
| Hill and Camacho, 2017 | United Kingdom | Assess effects of e-cigarettes on mortality and smoking prevalence | Simulation | Prior model, selected citations, federal statistics | People | Research team, experts |
| Hosking et al., 2013 | USA | Examine potential impact of colorectal cancer screening interventions; inform discrete event simulation model | Causal-loop diagram | Selected citations, expert interviews | People | Research team, experts |
| Inoue et al., 2022 | Japan | Estimate medical care expenditure (e.g., cancer care) | Simulation | Selected citations, federal statistics | Currency | Not specified |
| İrsoy et al., 2020 | Turkey | Examine trade-offs related to treatment of chemotherapy-induced neutropenia | Simulation | Prior models, selected citations | Cells | Not specified |
| Kalomoiri et al., 2017 | USA | Account for weather patterns in an estimate of cancer risk from benzene air pollution | Causal-loop diagram, simulation | Selected citations | Environmental contaminants | Not specified |
| Karanfil and Sterman, 2020 | USA | Illustrate hypothesis of overuse and fluctuations in routine medical screening | Simulation | Selected citations | People | Not specified |
| Kivuti-Bitok et al., 2014 | Kenya | Compare interventions for cervical cancer management | Simulation | Selected citations, cancer registries, expert opinion | People | Not specified |
| Liew, 2018 | USA | Evaluate impact of screening strategies on breast cancer diagnosis and mortality | Simulation | Cancer registry, federal statistics | People | Not specified |
| Lindberg et al., 2021 | Sweden | Improve methods of modeling patient referral to radiotherapy | Simulation | Patient data, selected citations | People | Not specified |
| McKnight and Finkel, 2013 | Germany | Estimate health risks from soil and groundwater contamination at a former military airfield; identify strategy for attenuating risk | Simulation | Selected citations, prior models | Environmental contaminants | Not specified |
| Mills et al., 2021 | USA | Identify connections between causes of smoking among racial/ethnic minority and low-income adults, anticipate effects of control policies | Causal-loop diagram | Literature review, participatory modeling sessions | Patient experience or behavior | Research team, participants |
| Palma et al., 2016 | USA | Assess benefits of prostate-specific antigen screening by replicating results of clinical trial to correct for noncompliance and contamination | Simulation | Trial data, federal statistics, selected citations | People | Research team |
| Ramsey et al., 2019 | USA | Characterize undertreatment of patients in hospital setting who smoke, identify possible leverage points | Stock-and-flow diagram | Participatory modeling sessions | People | Research team, participants |
| Saeed et al., 2021 | USA | Shift paradigm of cancer cell growth and treatment | Simulation | Selected citations | Cells | Not specified |
| Sant'ana et al., 2019 | Brazil | Assess effectiveness of managerial policies to reduce stress in oncology nursing teams | Causal-loop diagram, simulation | Participatory modeling sessions, clinic data | Patient experience or behavior | Research team, participants |
| Selya, 2021 | USA | Investigate potential of e-cigarette promotion to reduce cigarette smoking | Causal-loop diagram, simulation | Selected citations | People | Author |
| Shariatpanahi et al., 2017 | Iran | Assess effectiveness of WHO world awareness days for increasing disease awareness | Simulation | Prior model, selected citations; search engine data | Google search queries | Not specified |
| Tseng et al., 2019 | Taiwan | Assess cancer risk from chromium contamination; calculate necessary reduction | Simulation | Federal environmental data | Environmental contaminants | Research team |
| Williams et al., 2018 | USA | Identify reasons African American women delay or do not begin breast cancer treatment | Causal-loop diagram | Participatory modeling sessions | Patient experience or behavior | Not specified |
| Williams et al., 2016 | Global-focus | Develop conceptual framework of systems underlying trends in global cancer disparities | Hybrid CLD/SFD | Literature review | People | Research team, participants |
| Wong et al., 2012 | Canada | Develop conceptual model to improve emergency department wait times | Causal-loop diagram, SFD | Participatory modeling sessions | People | Author |

Abbreviations: CLD, causal-loop diagram; SFD, stock-and-flow diagram; WHO, World Health Organization
