# Supplementary material for: System dynamics modeling for cancer prevention and control: A systematic review
Source: PLoS One. 2023 Dec 1;18(12):e0294912. doi: 10.1371/journal.pone.0294912 (PMC10691687; doi:10.1371/journal.pone.0294912)
Supplement: S2 Table — (DOCX) [file pone.0294912.s005.docx]

**S4 Table – Quality assessment scores for included studies**

| **Author-date** | **Present clear objectives or purpose?** | **Identify information sources supporting model development** | **Clearly describe modeling process, including role of modeler(s) & participants** | **Involve stakeholders in model development, validation, and use** | **Calibrate model using real-world data*** | **Verify and validate model** | **Describe model structure using diagram(s) adhering to standard notation** | **Present clear outcomes and results using graphs, charts or tables*** | **Report model equations and parameter values*** |
| --- | --- | --- | --- | --- | --- | --- | --- | --- | --- |
| ***Simulation*** | | | | | | | | | |
| Alabdulkarim, 2018 | 5 | 1 | 0 | 0 | 0 | 0 | 5 | 2 | 3 |
| Chen et al., 2018 | 2 | 1 | 1 | 0 | 1 | 1 | 4 | 1 | 1 |
| Claeson et al., 2016 | 4 | 2 | 2 | 0 | 3 | 2 | 0 | 2 | 1 |
| Ehrenpreis and Smith, 2018 | 2 | 3 | 1 | 0 | 3 | 1 | 3 | 3 | 4 |
| Erten et al., 2016 | 2 | 2 | 1 | 3 | 2 | 0 | 0 | 1 | 1 |
| Heshmat and Eltawil, 2018 | 4 | 4 | 3 | 0 | 3 | 2 | 3 | 4 | 5 |
| Hill and Camacho, 2017 | 5 | 4 | 3 | 1 | 4 | 3 | 4 | 5 | 5 |
| Inoue et al., 2022 | 2 | 3 | 1 | 0 | 3 | 2 | 3 | 2 | 3 |
| İrsoy et al., 2020 | 5 | 5 | 2 | 0 | 5 | 5 | 5 | 5 | 5 |
| Kalomoiri et al., 2017 | 4 | 5 | 2 | 0 | 3 | 0 | 5 | 5 | 5 |
| Karanfil and Sterman, 2020 | 5 | 4 | 3 | 0 | 4 | 0 | 5 | 5 | 5 |
| Kivuti-Bitok et al., 2014 | 4 | 4 | 2 | 2 | 1 | 2 | 4 | 3 | 4 |
| Liew, 2018 | 3 | 1 | 1 | 0 | 0 | 0 | 1 | 2 | 3 |
| Lindberg et al., 2021 | 4 | 3 | 2 | 0 | 2 | 0 | 4 | 3 | 4 |
| McKnight and Finkel, 2013 | 5 | 5 | 3 | 0 | 5 | 2 | 4 | 5 | 5 |
| Palma et al., 2016 | 5 | 3 | 3 | 0 | 3 | 5 | 5 | 5 | 5 |
| Saeed et al., 2021 | 3 | 5 | 2 | 0 | 1 | 0 | 5 | 5 | 5 |
| Sant'ana et al., 2019 | 4 | 1 | 3 | 3 | 1 | 0 | 2 | 5 | 3 |
| Selya, 2021 | 4 | 1 | 2 | 0 | 2 | 3 | 5 | 4 | 4 |
| Shariatpanahi et al., 2017 | 2 | 3 | 2 | 0 | 3 | 0 | 4 | 3 | 4 |
| Tseng et al., 2019 | 3 | 2 | 1 | 0 | 1 | 0 | 3 | 3 | 5 |
| **Average for simulation studies** | **3.67** | **2.95** | **1.90** | **0.43** | **2.38** | **1.33** | **3.52** | **3.48** | **3.81** |
| ***Diagram-only*** | | | | | | | | | |
| Author-date | **Present clear objectives or purpose?** | **Identify information sources supporting model development** | **Clearly describe modeling process, including role of modeler(s) & participants** | **Involve stakeholders in model development, validation, and use** | **Calibrate model using real-world data*** | **Verify and validate model** | **Describe model structure using diagram(s) adhering to standard notation** | **Present clear outcomes and results using graphs, charts or tables*** | **Report model equations and parameter values*** |
| Ansah et al., 2019 | 3 | 5 | 4 | 5 | N/A | 0 | 5 | N/A | N/A |
| Beaulieu et al., 2022 | 3 | 4 | 4 | 3 | N/A | 5 | 5 | N/A | N/A |
| Brice et al., 2021 | 2 | 3 | 2 | 0 | N/A | 0 | 5 | N/A | N/A |
| Chen et al., 2019 | 4 | 4 | 5 | 3 | N/A | 0 | 5 | N/A | N/A |
| Hassmiller Lich et al., 2016 | 5 | 0 | 2 | 0 | N/A | 0 | 5 | N/A | N/A |
| Hosking et al., 2013 | 3 | 3 | 2 | 2 | N/A | 3 | 3 | N/A | N/A |
| Mills et al., 2021 | 5 | 5 | 5 | 4 | N/A | 5 | 5 | N/A | N/A |
| Ramsey et al., 2019 | 4 | 4 | 3 | 4 | N/A | 1 | 1 | N/A | N/A |
| Williams et al., 2018 | 3 | 5 | 5 | 5 | N/A | 4 | 5 | N/A | N/A |
| Williams et al., 2016 | 2 | 3 | 2 | 0 | N/A | 0 | 5 | N/A | N/A |
| Wong et al., 2012 | 3 | 3 | 2 | 3 | N/A | 1 | 5 | N/A | N/A |
| **Average for diagram studies** | **3.36** | **3.55** | **3.27** | **2.64** | N/A | **1.73** | 4.00 | N/A | N/A |

(*) Applied to simulation studies only
